# Technical and Clinical Progress on Robot-Assisted Endovascular Interventions: A Review

**DOI:** 10.3390/mi14010197

**Published:** 2023-01-12

**Authors:** Wenke Duan, Toluwanimi Akinyemi, Wenjing Du, Jun Ma, Xingyu Chen, Fuhao Wang, Olatunji Omisore, Jingjing Luo, Hongbo Wang, Lei Wang

**Affiliations:** 1 Academy for Engineering and Technology, Fudan University, Shanghai 200433, China; 2 Shenzhen Institutes of Advanced Technology, Chinese Academy of Sciences, Shenzhen 518055, China; 3 Shenzhen Raysight Intelligent Medical Technology Co., Ltd., Shenzhen 518063, China; 4 Shenzhen Engineering Laboratory for Diagnosis & Treatment Key Technologies of Interventional Surgical Robots, Shenzhen 518055, China

**Keywords:** robot-assisted vascular interventions, haptic feedback, control strategies, skill assessment, design taxonomies

## Abstract

Prior methods of patient care have changed in recent years due to the availability of minimally invasive surgical platforms for endovascular interventions. These platforms have demonstrated the ability to improve patients’ vascular intervention outcomes, and global morbidities and mortalities from vascular disease are decreasing. Nonetheless, there are still concerns about the long-term effects of exposing interventionalists and patients to the operational hazards in the cath lab, and the perioperative risks that patients undergo. For these reasons, robot-assisted vascular interventions were developed to provide interventionalists with the ability to perform minimally invasive procedures with improved surgical workflow. We conducted a thorough literature search and presented a review of 130 studies published within the last 20 years that focused on robot-assisted endovascular interventions and are closely related to the current gains and obstacles of vascular interventional robots published up to 2022. We assessed both the research-based prototypes and commercial products, with an emphasis on their technical characteristics and application domains. Furthermore, we outlined how the robotic platforms enhanced both surgeons’ and patients’ perioperative experiences of robot-assisted vascular interventions. Finally, we summarized our findings and proposed three key milestones that could improve the development of the next-generation vascular interventional robots.

## 1. Introduction

The causative factors of atherosclerotic plaques within the human body are not fully understood. However, these plaques’ appearance, development, spread, and subsequent effects within the body have been studied extensively [1]. For instance, coronary artery disease (CAD) develops when atherosclerotic plaques (fat, calcium, and inflammatory cells) accumulate within the coronary arteries, resulting in the thickening of arterial walls and the obstruction of blood flow to the heart muscle [2]. Besides, these plaques manifest in the blood vessels located in the upper and lower extremities. Early symptoms of CAD frequently include pain in the shoulders, arms, or chest; numbness; myocardial infarction; and even sudden death [3]. As a result, CAD significantly contributes to global disease burden, mortality, and rising hospitalization costs [4].

**Figure 1:**
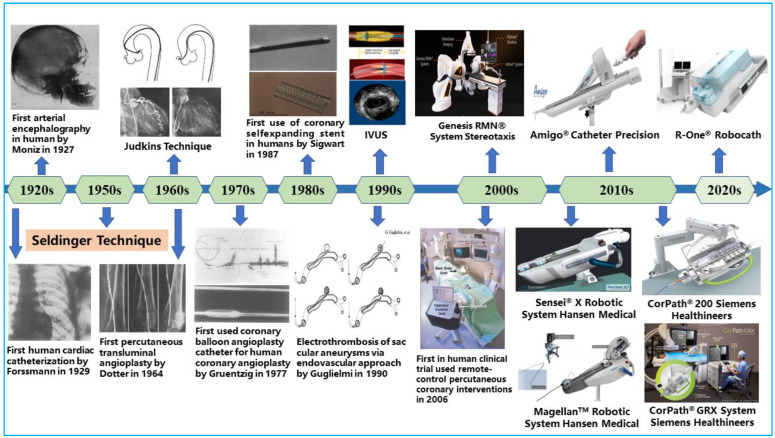
Key milestones in the field of endovascular interventional therapy.

Over time, open surgery, such as arterial bypass surgery, was performed to fix blocked coronary vessels to reroute blood flow to the heart. This treatment approach necessitates significantly large incisions in the patients’ chests, and it is commonly characterized by longer recovery time, risk of bleeding, potential infections at the incision site, and bad cosmesis [5]. Alternative therapies were required to treat vascular diseases because of the drawbacks of arterial bypass surgery. The development of interventional cardiology and the success of emerging technologies, however, were key factors in how vascular disease treatment has changed. As illustrated in Figure 1, the pioneering discovery of cerebral angiography by Egas Moniz in 1927 set the tone for today’s less invasive procedures [6]. Since then, different technological developments have been made to improve the diagnosis, treatment modality, and outcomes of minimally invasive procedures. At present, open-heart surgeries have been gradually phased out in favor of less invasive endovascular procedures that rebuild blood-flow pathways using endovascular tools [7]. This has become an effective treatment method for CAD, with patients benefiting from many of its advantages, including shortened recovery time and reduced perioperative risks [8].

Skilled interventional cardiologists perform PCI procedures by navigating flexible endovascular tools such as guidewires, catheters, and stents from a peripheral entry port to a target site in the coronary arteries using their motor, cognitive, and procedural skills [9]. In addition, fluoroscopic-based systems are integrated within the cath labs to provide a field of view during procedures, localize the size of lesions, and guide the catheter’s axial and rotary navigation while preventing damage to the blood vessels. With these approaches, enhanced patient outcomes are achieved compared to arterial bypass surgery, but at a cost to the interventionalist’s long-term health [10]. Primary steps to safeguard the interventionalist’s health from scatter-radiation risks during procedures such as the adorning of radiation shielding were mostly operator-dependent and without substantial effectiveness [11]. In addition, an increase in physical discomfort and orthopedic injuries were prevalent amongst the interventionalists, as reported in several studies [12,13]. The conventional procedures required interventionalists to have high angiographic precision for stent placement. However, there were occurrences of geographic misses, imprecise stent placement, and miscalculated stent length largely due to visual estimation by the interventionalist [14].

The inherent concerns associated with conventional endovascular procedures continue to limit the widespread acceptance of less invasive therapies. A recent way forward was found through the introduction of robotic systems for improved procedures and patient outcomes [15]. Based on this, several studies have shown that robot-assisted interventions can overcome the drawbacks of conventional vascular procedures [16,17,18]. For instance, it helps physicians to improve navigation accuracy and enhance stability and precision during catheterization, and is capable of eliminating imprecise navigation due to the operator’s hand tremors, thus helping to minimize intraluminal vessel damage. More importantly, it allows the interventionalist to operate from a safe distance, thereby minimizing the operator’s exposure to scattered radiation while still maintaining a substantial field of view during procedures [19]. This proof of concept and early-stage demonstration of robotic-system feasibility for endovascular interventions has accelerated the development of several robotic-system prototypes at the commercial scale over the last two decades [20,21,22]. The use of surgical robots for different interventions has gradually increased in the operating room due to advantages such as operation speed, navigation precision, dexterity, and action reproducibility when compared with expert human performance [23]. Similarly, within the research domain, research-based prototypes of robotic systems for PCI procedures have been developed and are being scaled up for commercial viability [24,25,26,27,28]. Furthermore, the safety, feasibility, and clinical adoption of existing robotic systems for neurovascular interventions, cardiovascular interventions, peripheral vascular interventions, and electrophysiological interventions have been reported in several studies [29,30,31,32,33]. These interventional domains formed the bases for the advocacy of the adoption of robot-assisted endovascular interventions.

This study aims to explore the current progress and technical challenges of the existing robotic systems across these domains, with an emphasis on selected endovascular procedures. It should be noted that despite the proven safety and usability of robotic systems for endovascular procedures, it is posited that their limited application areas, incompatibility with over-the-wire surgical instruments, and the absence of force feedback or haptic perception are obvious limitations. Furthermore, there is a need for an increased autonomy, enhanced machine intelligence, potential radiation-free medical-imaging modalities, and improved design, techniques, and instrumentation with micrometer accuracy. Hence, this review is designed to carefully analyze the state-of-the-art approaches and present challenges of various segments of vascular interventional robots (VIRs). In addition, suggestions on the future perspectives for interventional robots both at the research and commercial levels are outlined within the Conclusion section. The above demonstrates the technical issues that need to be addressed to expand the commercialization and clinical application of VIRs. Thus, as our motivation, we position this review study to provide technical and clinical progress on robot-assisted endovascular interventions. An analytical overview of the present gains and current challenges of VIR are presented via four themes, such as the design innovations of robotic technology, guidance systems and robotic-manipulation control, perception systems, clinical applications, and evaluations.

The remaining parts of this review are organized such that Section 2 presents the key technologies and application areas of VIR and current strategies to enhance VIR benefits to patients, and Section 3 presents some progress on the clinical adoption and evaluation of robot-assisted endovascular interventions. A discussion on the outlook and current technical challenges hindering the widespread adoption of the robotic technology is presented in Section 4. This includes further routes for research, engineering, and developmental studies in the domain of robot-assisted endovascular interventions, and the conclusions of the study.

## 2. Key Technologies and Application Areas of Vascular Interventional Robots

Recent advances in the fields of robotics, sensors control, computer vision, and artificial intelligence have fueled rapid growth in the area of medical robotics [34,35,36]. Over the last few decades, several types of endovascular robots have been developed. Typically, VIR combines sensing, actuation, and tool-clamping mechanisms with a wireless communication protocol, control system, and user interface for robust endovascular procedures. Such procedures require efficient collaboration between the surgeon and the robot to achieve safe, precise, and dexterous tool movement within the patients’ blood vessel. In addition, control and safety strategies are implemented within the master–slave platform coupled with image-based guidance systems to minimize operative risks during procedures. Furthermore, the tool–vessel contact force and haptic feedback are essential for closed-loop control modeling and potential autonomous navigation based on increased machine awareness and operation safety during catheterization [37,38]. These essential areas for safe robot-assisted vascular interventions are discussed elaborately in the subsequent sections.

### 2.1. Driving Mechanisms and Teleoperation Setups

#### 2.1.1. Classification by Driving Mechanisms

**Figure 2:**
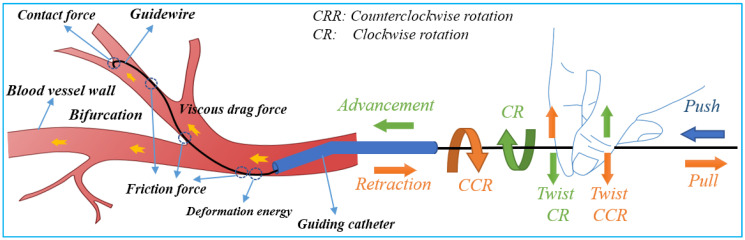
Schematic of guidewire manipulations and force analysis on the surgeon’s fingers.

Safe navigation of flexible endovascular tools through blood-vessel paths for stent and balloon delivery requires that surgeon uses their forefinger and thumb skillfully [39]. These intelligent-hand defter procedures requires stimuli interplay of kneading the endovascular tools to manipulate the thin, long, and flexible tools back and forth along blood vessels. When a bifurcation is encountered, the two fingers are rubbed up and down in relation to each other to change the direction of the guidewire tip in order to pass through the bifurcation. The schematic diagram of guidewire manipulations and force analysis by the interventionalist’s thumb and index fingers is shown in Figure 2. Based on the manipulation modes illustrated in Figure 2, it can be seen that the motion of the robot requires at least three actions—clamping, translation, and rotation for two degrees of freedom (2-DOF) of endovascular tool motion (axial and rotary) within the blood vessels.

**Figure 3:**
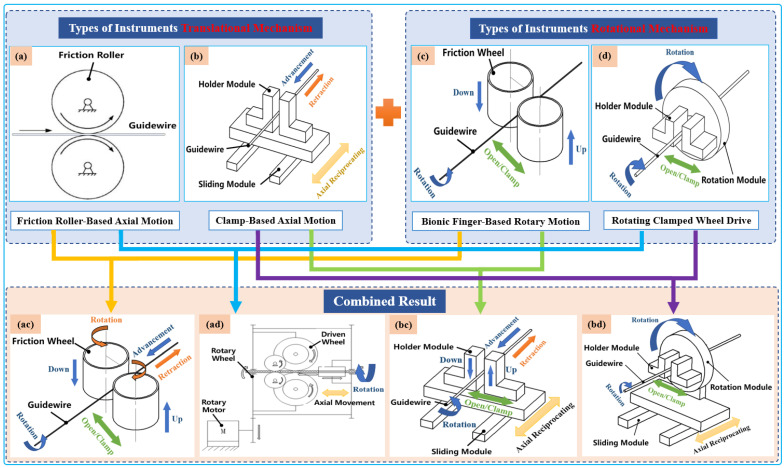
Classification schematic diagram of instruments’ driving mechanisms. Types of translational instrument mechanisms: (**a**) friction roller rotating along its axis to translate guidewires; (**b**) holder module clamp guidewire axial reciprocating motion on the sliding module. Types of rotational instrument mechanisms: (**c**) two bionic clamp fingers move linearly in the opposite direction to rub the guidewires; (**d**) holder module clamp guidewires rotated on the rotation module. Combined result: (**ac**), (**ad**), (**bc**), and (**bd**) are the result of permutation and a combination of translational instrument mechanisms with rotational mechanisms to realize the simultaneous advancement and rotation of guidewires.

Typically, two types of axial-drive mechanism are utilized in VIR for highly precise linear motion, which is then combined with two rotating mechanisms. Both combinations can achieve four different modes of axial and rotary mechanisms that can generate simplified 2-DOF tool motion, as illustrated in Figure 3. Currently, VIRs mostly use an arrangement of one of these axial and rotary mechanism to drive the guidewires or catheters intuitively during procedures [40,41]. Therefore, an overview of these modes is presented below.

(1) Translational Tool Mechanisms

The axial mechanism for endovascular-tool motion can be categorized as either friction roller-based or clamp-based mechanisms, as shown in Table 1. Friction roller-based mechanisms consist of a pair of friction rollers that axially moves the guidewire/catheter forward or backward through the friction that emanates when the rollers are pressed against each other. This mechanism has the advantages of compactness, minimal size requirement, and convenient tool clamping and disinfection. However, a primary drawback is the occurrence of slippage of the friction-wheel-driving method, which can affect the control accuracy when the two friction wheels are not parallel. In contrast, the clamping-based mechanism is designed to imitate the surgeon’s gradual guidewire-delivery process and to reduce the length of the axial mechanism. This axial reciprocating motion mechanism comprises a clamping device and slider rail driven by a motorized linear actuator. The motorized linear actuator facilitates the slider rail’s return to its home position after a full stroke without any backward movement of the tools while the clamp also sustains a firm grasp of the tool. After this, the clamp is released and the slider rail moves forward for continuous translation of the guidewire or catheter until the desired catheterization length is reached. This mechanism ensures reliable guidewire propulsion accuracy can be achieved and facilitates the measurement of the guidewire’s resistance within the vessel. However, the disadvantage of the clamp-based mechanism is that the mechanism occupies a large volume of space.

(2) Types of Rotational Instrument Mechanisms

Rotational mechanisms applied in VIR for generating rotary movement of endovascular tools generally involve the use of bionic finger-based and rotating clamped-wheel mechanisms, as shown in Table 1. The bionic finger-based mechanism can be described as consisting of two parallel claws that imitate the surgeon’s thumb and index fingers. When these claws press on each other, they serve as a clamp and can rotate the guidewire. The advantages of this structure are that it is convenient to arrange the tools and to carry out the sterilization operation. However, the rotational angle is limited and the accuracy cannot be fully ascertained. In contrast, rotating clamped-wheel drive mechanisms are usually driven by mechanism such as gears and synchronous belts to realize the guidewire rotation. It can ensure that the guidewire rotates at any angle with high accuracy, but the drawback is that the structure of the mechanism makes it difficult to carry out adequate tool sterilization.

Existing VIR systems frequently combine these two basic mechanisms for tool translation and rotation during procedures. This includes commercial endovascular robots such as the CorPath^®^ GRX system (Siemens Healthineers, Pennsylvania, USA), which adopts a friction wheel combined with a rotary wheel for 2-DOF guidewire/catheter movement. In contrast, the Magellan system (Auris Surgical Robotics, California, USA) adopts the friction wheel for rotational and axial tool motion [42]. Furthermore, Bian et al. [43] designed two bionic fingers that utilize a friction wheel to imitate the surgeon’s manipulation skills for catheter navigation. However, Wang et al. [44] installed four manipulators that mimic physician’s four fingers to enable tool delivery based on the four wire ropes. The authors installed a motor gear to facilitate independent tool rotation by each manipulator.

Despite the usage and feasibility of these mechanisms, the need to develop intuitive and improved driving mechanisms for VIR still exists. For example, Shen et al. [45] analyzed the demerits of the above-mentioned translation mechanisms, i.e., friction and continuous approaches, and combined these mechanism advantages to develop a hybrid translational mechanism using friction and a clamping device for axial tool motion in a robot-assisted neurovascular intervention. In addition, Choi et al. [46] utilized multiple friction wheels to form friction-wheel groups as a method to overcome the drawbacks of the slippage effect in friction roller-based drive mechanisms. The outcome of this study showed that the friction-wheel grouping approach resulted in an improvement to the robotic system’s translational mechanism.

#### 2.1.2. Teleoperation Setup

In order to reduce occupational hazards such as radiation exposure and orthopedic injuries that surgeons experience during endovascular procedures, VIRs have been designed with capabilities for remote manipulation. This usually involves the use of bedside instrument tool-driving mechanisms (slave device) and a control interface (master device) that the operator handles at the cockpit station. For ease of usage at the control station, interventionalists often visualize the surgical scene on multiple display units and access the master device for tool manipulation. This setup serves its purpose by protecting the surgeon from scattered radiation and grants them ergonomic comfort to cannulate the patients’ blood vessel under imaging guidance [47]. At present, the design of the master–slave robot platform includes isomorphic and non-isomorphic teleoperation setups, as highlighted in Table 1 and described below.

(1) **Isomorphic setup:** An isomorphic teleoperation involves using more ergonomic master interfaces that allow surgeons to replicate their natural hand-movement patterns during interventions. In this setup, the master-and-slave systems have similar structural and functional designs. Thus, the actions commands issued on the master side are homogenously replicated in the slave-side device. This makes slave devices exhibit interventionalists’ hand-and-finger dexterity for fine motor-based tool manipulation. Isomorphic setups are new in the endovascular intervention domains. However, recent studies have shown that it can reduce surgeons’ learning curve since they can directly utilize their natural catheterization skills. The isomorphic design by Thakur et al. [25] directly utilizes a real input catheter as the master device and a sensor to record the catheter’s motion while the slave device replicates the master motion to drive a catheter inside the vessel. Similarly, Payne et al. [48] developed a novel master–slave force-feedback system that conforms to a doctor’s natural operating habits and ergonomics. The interface of the same configuration is in line with the intuitive operation of doctors, which is easier to understand and learn. More and more isomorphic platforms have been developed and utilized in recent studies [49,50,51].
(2) **Non-isomorphic**: Non-isomorphic teleoperation design is the earliest and most common approach utilized in robot-assisted minimally invasive interventions. In this mode, a significant difference exists in between the structural design of master-and-slave devices in robotic-platform structural design. Specifically, the robotic setup has master-and-slave control interfaces with a unique design and tool-handling schemes. Currently, most of the commercial robotic systems used for endovascular interventions are generally non-isomorphic. For instance, the control interfaces in CorPath^®^ GRX and CorPath^®^ 200 robotic catheter systems are based on joysticks and touch screens [52]. The typical designs of the CorPath interfaces allows surgeon to manipulate endovascular tools like guidewires with one hand and operate other tools such as the balloon/stent catheter with the other hand. Similarly, in the Amigo^®^ system (Catheter Precision, Inc., Ledgewood, NJ, USA), another major commercial interventional robot used for electrophysiological interventions, the master device is designed as a wireless remote controller for catheter manipulation. The system is able to reproduce linear catheter motions, rotary motion, and tip deflection all issued by the appropriate buttons with one hand on the master device [53]. Although this controller system has an intuitive input method, the design and form are essentially different from the slave robotic platform. Relatedly, some other non-isomorphic setups involve the use of commercial 3-DOF haptic devices as the master-side platform. Typically, Ma et al. [54] and Shen et al. [45] selected Omega (Force Dimension, Nyon, Switzerland), a parallel manipulator capable of producing force feedback to the operator, as the master interface. The commercial controllers are generally adaptable to existing robotic systems. However, customizing them for tool-delivery mechanisms is sometimes difficult.

### 2.2. Guidance Systems and Robotic Control Scheme

#### 2.2.1. Image-Based Guidance Systems

The exact navigation of endovascular tools within the blood vessels is a key aspect of minimally invasive interventions. During these procedures, the surgeon aims to maintain a continuous mental grasp of the endovascular tool’s actual position in order to steer the guidewire or catheter safely within the vasculature. However, to achieve this, image-based guidance systems are designed to complement VIRs. These systems visualize the catheter’s position non-invasively, localize coronary lesions, and help to minimize the occurrence of ruptures during procedures. For endovascular interventions, a number of catheter-guidance technologies have been developed and adopted in the cath lab. Broadly speaking, these can be divided into extravascular imaging-system modalities, such as digital subtraction angiography (DSA), computed tomography (CT), magnetic resonance (MR), and ultrasound (US), and intravascular imaging modalities, such as intravascular ultrasound (IVUS) and optical coherence tomography (OCT).

Currently, DSA is the imaging technique used most frequently by interventionalists because of its higher spatial and temporal resolution. With the DSA system, the physician can choose the interventional path or pinpoint the lesions’ size and distribution based on anatomical knowledge and real-time 2D angiography and fluoroscropy sequences of vessel imaging. The 2D-imaging technique is quick and clear enough to meet the needs of real-time intraoperative vascular imaging; however, it lacks 3D spatial information [55]. In contrast, 3D vascular images can be reconstructed using CTA. Typically, to compensate for the lack of 3D spatial information in the 2D image during operation, a preoperative vascular model is constructed prior to the procedure and registered with the 2D image taken in real-time during procedures. This offers an intuitive visual reference for precisely tracking the placement of surgical tools inside vessels for diagnosis and treatment. However, the disadvantages of 3D CTA include the low signal-to-noise ratio, large radiation dose, insufficient real-time performance, and presence of artifacts [56].

Compared to fluoroscopic imaging, MRI systems can produce both 2D and 3D images (MRA—magnetic resonance angiography), have high contrast to soft tissue, and pose no radiation risk. However, due to the presence of breathing and heartbeat movements, as well as anatomical factors like vascular torque and venous structure overlap, the image quality depreciates, resulting in artifacts and other defects present within the images. In addition, the patient is in a small, closed-loop scanner during MRA, which presents a significant challenge to the surgical robot’s structural design and magnetic compatibility [57]. US imaging technology can be utilized to assess the location, size, and shape of tissues and organs as well as the extent of lesions. It also has a significant effect on soft tissues and can provide depth information. It can be used in addition to 2D fluoroscopy images and is non-radiative, portable, and easy to use. However, its use in vascular interventional surgery is constrained by the inability to accurately visualize the catheter or guidewire’s spatial pose, which is a drawback [58].

Recent years have seen a rapid development of a number of intravascular imaging techniques that can navigate through smaller vascular lumen prior to and post-stent implantation and can be used to evaluate the plaque coverage, stent placement, and expansion degree. IVUS and OCT are the intravascular imaging techniques that have received the most research to date. IVUS creates images using high-frequency ultrasound to assess the degree of vascular stenosis and identify bifurcations and calcified lesions, among other things. These images reflect the layered structure of vascular tissue. However, the primary drawback of IVUS is its low resolution, which makes it challenging to determine the fibrous cap and hyperechoic plaques’ exact thicknesses. The use of IVUS in small vessels and severely stenotic vessels is also limited by the size of the ultrasound probe. In contrast, OCT can detect and categorize plaques more precisely than IVUS because of its higher spatial resolution and imaging-acquisition speed, which can be up to 10 and 40 times higher, respectively. Its disadvantage is that when blood flow is present, it has lower imaging quality and tissue penetration [59]. However, by combining the penetration of IVUS with the high resolution of OCT, many researchers have developed a hybrid IVUS–OCT probe to improve the accuracy of intravascular imaging and navigation [60]. Beyond this, some other studies have fused IVUS/OCT images and angiographic images to create 3D vessel reconstruction and to determine the position and direction of the catheter, which could open up alternative intraoperative 3D navigation [61].

#### 2.2.2. Robotic-Control Scheme

In robot-assisted endovascular interventions, the master–slave teleoperated system utilizes a control loop involving the human operator. Typically, the master controller deduces the surgeon’s actions and transfers corresponding input signals to the slave controller. The latter outputs appropriate control signals to the linear drive system for axial and rotational tool movements. However, in real systems, the slave robot’s linear drive mechanism experiences motion lag resulting in a slight deviation from the master’s input motion commands. This difference caused by tool–tissue friction, communication delay, nonlinear disturbances such as hysteresis and backlash, and other effects generally leading to inaccurate master–slave position trajectory and fluttering, which may cause tool drift and vascular perforation. Therefore, control systems are essential to minimize this effect and should have desirable characteristics such as high precision, fast response, tremor elimination, and surgical-safety early warning. Thus, several feedforward and feedback control-system implementations exist for different master–slave robotic systems.

Open-loop control using position-control mode utilizes a feedforward controller, which aims to provide precise positioning without reliance on the slave robot’s output feedback. This is often a common control strategy [62]. However, feedforward systems could be simplistic, inaccurate, and unreliable for motion-control tasks essential for robot-assisted catheterization. An example of the feedforward control systems for VIR include that in Thakur et al. [25], where the authors utilized a constant scaling factor for master–slave motion mapping. Although this method could improve the master–slave position accuracy, it is a non-adaptive approach that could often require retuning and the occurrence of errors for unbounded intervals. Whereas the former study was based on constant scaling, Feng et al. [63] utilized an adaptive motion-scaling method. This was deployed into the master–slave device for adaptive tool navigation during robot-assisted vascular interventions. The authors evaluated the master–slave position deviation and introduced different scaling factors for proportional, reduced, or magnified input-to-output commands for different segments of a catheterization stroke.

However, the practicability of the techniques in real-time systems poses concern such that closed-loop control systems have received increased attention for position tracking and error compensation to improve the accuracy of the catheter/guidewire insertion and navigation within the vasculature. This includes position control, force-based control, motion compensation, image-based navigation, and learning-based control schemes based on deep-learning and reinforcement-learning algorithms. These control methods have been applied to VIRs remarkably.

The PID controller is the most commonly used control approach in VIRs and consists of tuning proportional, integral, and differential gains for smooth motion during robotic catheterization. Some configurations of PID-based controllers have been applied within this domain for master–slave position-error compensation. For example, in Ref. [44], the authors utilized the PID controller within their master–slave platform for control of the endovascular tool’s axial and rotational movements during robot-assisted peripheral vascular intervention. The PID control gains were utilized to determine matching step values for the linear drive actuator to obtain uniform input–output position commands. Overall, the controller compensated for the initial position error; however, the final error was around 0.5 mm. Similarly, Sankaran et al. [24] developed a cascade controller, which integrated an adaptive input shaper and the PID controller to achieve closer master–slave position tracking during robot-assisted catheterization. The study used guidewire resistance force as a measure of proximal feedback to enhance patient safety during catheterization. However, conventional PID controllers have some inherent limitations, including the occurrence of noise in the derivative gains, poor real-time performance, and consistency required for smooth catheterization during robotic interventions. Based on this, several research groups have proposed cascaded configurations of fuzzy–PID controllers employing fuzzy rules to fine-tune the linear parameters of the PID control gains dynamically.

For instance, Song et al. [64] proposed the position control of a master–slave robot using intelligent fuzzy–PID controllers capable of online PID control gains and fuzzy-rule tuning. In addition, Yu et al. [50] developed a dual fuzzy–PID controller for online control-parameter tuning and interference removal in a VIR. Furthermore, Guo et al. [65] implemented fuzzy–PID controllers within a slave robotic device to improve the slave robot’s position-tracking capability with the issued master command. Compared with the conventional PID controllers, fuzzy–PID does not require an accurate methodical model and can better deal with time-varying, non-linear hysteresis problems, with good robustness and fast response time. However, the control rules are non-adaptive and require more time to be appropriately designed [66].

Besides classical controllers, Wang et al. [28] designed an adaptive sliding-mode controller for a master–slave system to resolve the nonlinear and uncertain disturbances that the catheter/guidewire encounters within a linear drive system, thereby reducing the deviation from the input motion command and improving the response speed and accuracy of the control system. The controller had a better performance than PID, with a final error between 0.07 and 0.3 mm. In contrast, Omisore et al. [67] proposed and developed an adaptive neuro-fuzzy control system in a 2-DOF robotic catheter system for backlash compensation and force control using the robots’ kinematic parameters. The in-vitro experiments validated the neural network model’s aptness for improving position-tracking error within the slave robot; hence, a final error of 0.4 mm was obtained. Recently, Zhou et al. [68] adopted an auto-disturbance rejection-control approach for a VIR. The model’s working principle hinges on tuning four subcomponents to control the target displacement and improves the real-time position-tracking accuracy of an endovascular tool. In comparison, the control accuracy and response speed of the control strategy highlighted above were much better than the conventional PID control method. Overall, these models yielded good position control, and they could better handle the input–output dynamics in the master–slave setup. Furthermore, the studies show that the model offered fewer errors compared with PID-based controllers.

Despite the above-mentioned merits, machine-learning and artificial-intelligence algorithms are being utilized in newer VIR control models. These AI-based control models can achieve better tool catheterization and human–machine collaborative control. For instance, Ma et al. implemented a multi-layer neural-network model to tune PID parameters effectively and to improve the accuracy of the slave robot’s axial displacement. The study compared the MLP-tuned PID controller with conventional PID, and the result indicates that the neural-network-tuned controller had a better performance than the traditional PID control system [69]. Similarly, Wu et al. [70] utilized the long short-term memory network (LSTM) to model the hysteretic effects of a unidirectional robotic catheter and to track the position accuracy of its tip under different twist angles using the catheter’s kinematic parameters. Recently, Omisore et al. [66] proposed a deep reinforcement-learning model that could adaptively tune PID control gains for responsive tool tracking during robot-assisted PCI. The model evaluated via in-silico experiments achieved high tool-position accuracy with an RMS error of 0.003 mm. An advanced strategy published by Kweon et al. [71] shows that imitation or reinforcement learning can be directly designed for autonomous navigation of endovascular tools. In addition, Karstensen et al. [72] adopted deep deterministic policy gradients with hindsight experience replay for a learning-based control of guidewire navigation in a robot-assisted peripheral vascular-intervention study. The reinforcement-learning-based model was not reliant on human demonstration examples and had a 100% success rate for simulation-based studies. However, a lower precision was reported for the ex vivo study. Generally, in vivo applications of learning-based control models are still lacking.

So far, most studies have focused on master–slave control accuracy and safety, and the emphasis on patient safety and excellent control modes has led to the evolution of different control models with their respective advantages. However, at present, there is no widely accepted control method approved as the standard for position accuracy in VIR. Each control method is analyzed based on its strengths and limitations; however, in the future, the realization of a consensus could be possible. In conclusion, a list of contemporary robotic systems developed and commercialized for endovascular interventions is presented in Table 1. These vascular robotic systems are categorized based on the key technologies discussed above with application areas covering endovascular interventions.

**Table 1:** Summary of related robotic systems for vascular intervention.

Group	Driving Mechanisms (Translation/Rotation)	Teleoperation Setup	Control Scheme	Perception/Feedback	Guidance Systems	Application Areas
CorPath^®^ 200&GRX [73,74,75]	Friction roller-based/Rotating clamped wheel	Non-Isom.	Position and velocity	Obstacle feedback	DSA	PCI PVI NVI
Magellan^TM^ [42,76]	Friction roller-based/Friction wheel-based rotation	Non-Isom.	Position and velocity	Haptic	DSA/CT	PVI
Guo et al. [26,77]	Clamp-based/Rotating clamped wheel	Non-Isom./Isom.	Position and force	Haptic/Proximal force	DSA	PCI
Wang et al. [28,78]	Clamp-based/Rotating clamped wheel	Isom.	Position and force	Haptic/Proximal force	DSA	PCI
Wang et al. [40,79]	Friction roller-based/Bionic finger-based rotary	Non-Isom./Isom.	Position and velocity	N/A	DSA	NVI
Wang et al. [44,80]	Clamp-based/Rotating clamped wheel	Non-Isom.	Position and velocity	N/A	DSA	PCI PVI
Cha et al. [27,81]	Friction roller-based/Rotating clamped wheel	Non-Isom.	Position and force	Haptic/Proximal force	DSA	PCI
Choi et al. [82]	Friction roller-based/Bionic finger-based rotary	Non-Isom.	Position and velocity	N/A	DSA	PCI
Yang et al. [48,83]	Friction roller-based/Rotating clamped wheel	Isom.	Position and force	Haptic/Distal force	MRI	PCI
Tanimoto et al. [84]	Friction roller-based/Rotating clamped wheel	Isom.	Position and force	Haptic/Distal and Proximal force	CT	PCI
Omisore et al. [67,85]	Clamp-based/Rotating clamped wheel	Isom.	Position and force	Haptic/Proximal force	DSA	PCI
Bian et al. [41,43]	Friction roller-based/Bionic finger-based rotary	Isom.	Position and velocity	N/A	N/A	PCI
Zhou et al. [86]	Clamp-based/Rotating clamped wheel	Non-Isom.	Position and force	Proximal force	N/A	PCI
Li et al. [87]	Clamp-based/Rotating clamped wheel	N/A	Position and velocity	N/A	IVUS-OCT	PCI
Langsch et al. [88]	N/A	N/A	Position and velocity	Proximal force	US	PCI

Abbreviations: PCI—percutaneous coronary intervention; PVI—peripheral vascular intervention; NVI—neurovascular intervention; N/A—not applicable.

### 2.3. Perception Systiems

During endovascular procedures, the operator should perceive the tactile feedback of the force with which the endovascular tool is steered within the vasculature during robotic catheterization. However, current VIRs are only able to detect clamping and proximally applied force. However, most existing VIRs are primarily limited by their inability to sense and provide feedback on the interactive tool–vessel contact force during procedures. The absence of tool–vessel contact force increases the possibilities of operative risks such as thrombosis and vascular perforation that could arise from reliance on visual feedback, poor hand–eye coordination, and its impact on the mental and cognitive load of the interventionalist during procedures [89]. To resolve this lack of force feedback, researchers are exploring different approaches to measure the distal and proximal force in VIRs. This will enhance surgeon’s telepresence and feedback on applied manipulation force during robotic procedures.

#### 2.3.1. Force Feedback

Proximal and distal force measurements are essential in VIR safety and enhanced operation strategy during procedures. Whereas the former is measured nearer to the operating end of the catheter via off-the-shelf force sensors, the latter is measured towards the farthest point along the catheter’s length and is of more concern to the interventionalist. The distal force measures the contact force between the tip of catheter and the vessel walls and requires a miniaturized sensor that should be embedded within the coronary catheter. Therefore, these sensors’ dimensions, resolution, biocompatibility, measurement range, and accuracy are critical characteristics [90]. Both vascular and electrophysiological interventions have applications of force-measuring catheters. In electrophysiological interventions, for instance, force-measuring catheters are used to determine the catheter’s electrode contact with the myocardium. This helps in preventing mounting excessive contact force when creating scars on abnormal heart tissues. This technology has attained commercialization, and some available products are the TactiCath^®^ Catheter (Endosense SA, Geneva, Switzerland) and IntelliSense^®^ System. The contact-force catheter of TactiCath is a steerable 7-F radiofrequency-ablation catheter that integrates a force sensor at the distal end and measures the contact force between the catheter tip and heart tissue [91]. To improve stability under dynamic conditions during vascular interventions, a force-measuring catheter usually integrates the strain gauge or pressure-sensitive rubber and encapsulates the active part of the catheter with biocompatibility. For example, Guo et al. [92] arranged the pressure-sensitive rubber-sensing units in the front-end array of ducts and encapsulated them to detect the forces of different nodes. Omisore [67], Payne [48], and Wei [93], amongst many, designed catheters with over-the-wire force sensing (i.e., using strain gauge) to measure intravascular contact forces. Although resistive and strain sensors have excellent linearity, hardware such as circuits and metal substrates are vulnerable to electromagnetic interference during surgery, and the size is too large to be suitable for narrow vascular cavities [94]. FBG sensors have the advantages of small size, no electromagnetic interference, and high sensitivity. Recently, researchers integrated FBG-based fiber-optic force transducers into the catheter to solve the problem of size and electromagnetic interference [95]. He et al. [96] developed a catheter integrating four FBGs for intravascular force measurement and temperature compensation.

**Figure 4:**
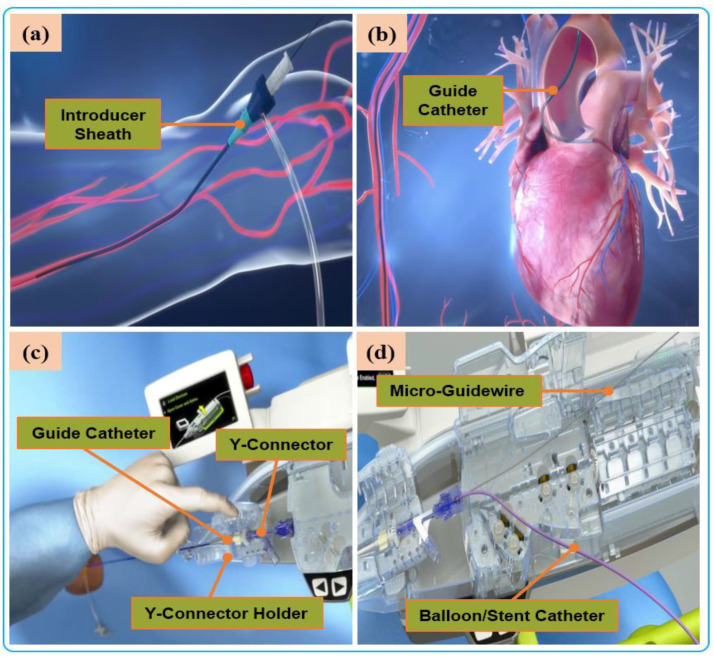
Manual procedure: (**a**) surgeon makes a small incision in the femoral/radial artery and insert an introducer sheath into the body to establish access for external instruments. (**b**) The guide catheter is hung on the coronary artery opening. (**c**) The guide catheter is attached to the Y connector and installed on the Y-connector holder to establish a stable track for device delivery. (**d**) A micro guidewire and balloon/stent catheter is loaded close the shell.

Although distal forces are important during electrophysiological ablation, the natural sense of touch felt by the surgeon is the proximal force in manipulating surgical instruments during traditional vascular interventions. The four intravascular force components constitute a complete proximal force: the viscous force of the blood, the collision force at the front end of the instrument, the friction force between the blood vessels, and the potential elastic force of the guidewire [20], as shown in Figure 4. The proximal-force measurement is usually carried out in a sensor-based manner, in which a high-precision and high-resolution force sensor is installed on the device-delivery mechanism to measure the contact force between the device and the blood vessel. However, there are also differences in the sensor installation and the force-measurement method. For instance, Yang et al. [97] developed a guidewire force-measuring mechanism based on the lever principle. The pressure sensor is installed on the propulsion finger, and the resistance is transmitted to the force-measuring mechanism through the guidewire. As the lever amplifies the signal, the force-measuring mechanism is unaffected by the disturbance in the transmission structure. Bao et al. [98] installed the force sensor on the clamping side of the guidewire, use the linear bearing to reduce the interference of friction on the guidewire resistance in the transmission process, and designed a multi-level safety-control strategy according to the force level to reduce the risk of operation. Similarly, Zhou et al. [86] and Wei et al. [93] use the force-measurement method of installing a sensor in the guidewire clamping part to measure and evaluate the resistance of the guidewire to improve the safety control of the robot. Sankaran et al. used the current of the drive motor to estimate the resistance of the guidewire and used the double-layer optimization method to calibrate [24]. The above sensor-based proximal force-measurement methods are most sensitive and generally able to measure the approximate value of resistance when the translational speed is low, the inclination angle is minimal, and the friction of the mechanism is very small [99]. However, during the robot’s operation, the resistance value obtained proximally is often inaccurate and susceptible to interference, mainly arising from weak resistance caused by either the frictional force in the actuator, the inertia force, or the presence of jerk in the linear drive system [100,101].

In addition to sensor-based force-measurement methods, model-based force measurement is also available. The force of the device in the model is calculated and estimated by combining sensor boundary conditions, real-time imaging, vascular anatomy, and the device model [102]. Therefore, the mechanical model of the guidewire or catheter must be accurate, and the solution process must be in real time, accurate, and stable. There are many modeling methods: the continuum mechanics model, many-body dynamics model, differential geometry model, and particle-based model. Due to the low cost and low external interference, model-based methods have gradually become a new direction of force-feedback research [103]. However, the disadvantage is that the nonlinear integration of the dynamic model may be numerically unstable and computationally expensive, so it is not easy to realize.

#### 2.3.2. Haptic Perception

Haptic perception is to transmit the feedback force between devices and vessels to the surgeon’s operation side through the control system. Through human–computer interaction, the interventionist can feel the resistant force of the endovascular tools, thus providing force perception and feedback, which can reproduce the haptic perception of vascular surgeons in traditional surgery and reduce the risk of surgery [104].

Amongst the commercially available master manipulators with force-feedback capacity, the Phantom Omni (Sensable Technologies, Wilmington, MA, USA) and Geomagic Touch X (3D Systems, Rock Hill, SC, USA) are the first to be used for catheter-insertion control and tactile feedback [105]. Their main structure is a series-control mechanism designed based on motor current to output torque and braking force. Another device, Omega Haptic Devices uses a Delta-based parallel-control structure. It is widely used in the master manipulator of medical robots due to flexible control, high spatial resolution, and sensitive force feedback [106]. However, these commercial robot-operated main hands have a much longer learning curve when it comes to surgical interventions that go against the surgeon’s traditional surgical skills [89]. Therefore, to better adapt to the clinical needs, researchers have conducted in-depth research on the intuitive master–slave isomorphic force-sensing control mechanism. Yan et al. [97] designed a master–slave isomorphic surgical robot. The master force sensing realized surgeons’ precise perception of the slave guidewire force through the interactive predictive control of the motor, torque sensor, and guidewire resistance. Guo et al. [26] proposed the haptic feedback technology based on magnetorheological fluid to measure the operator’s movements and provide haptic force. The in vitro experimental results showed that the haptic feedback based on catheter was helpful for improving intubation skills and reducing the cognitive workload of operators. Li et al. [107] used similar principles to develop a collaborative control platform based on magnetorheological fluid and hydrogel modeling that could replicate operator movements while providing haptic force feedback. Payne et al. [48] developed a “hands-on” master–slave control system that used voice-coil motors to provide force feedback to the operator. Through body-membrane experiments, it was verified that the contact force between the catheter and the tissue could be significantly reduced. Commercial master manipulators provide steady force feedback but are a challenge for doctors to learn their skills. However, a master control mechanism with an isomorphic master–slave limitedly supports surgeons’ surgical habits. Thus, some challenges such as unstable force feedback, high friction force, and inability to eliminate doctors’ hand tremor needs further study.

### 2.4. Application Areas of Vascular Interventional Robots

VIRs are categorized based on their application areas. Robot-assisted vascular intervention can be divided to into four, which include robot-assisted percutaneous coronary intervention (R-PCI), robot-assisted peripheral vascular intervention (R-PVI), robot-assisted neurovascular intervention (R-NVI), and robot-assisted electrophysiological intervention (R-EPI). Three of these specialty domains (R-PCI, R-PVI, R-NVI) possess some similarities in the way the procedures are carried out except that the location of the blood vessels differs from one another, such as the heart coronary, the lower extremity, and the head region. Nevertheless, commercial robotic systems were initially designed as sub-specialty systems targeted at R-PVI or R-PCI. This includes the Hansen Magellan system for R-PVI and the CorPath robotic systems and R-One robotic System (Robocath Inc., Rouen, France) for R-PCI. However, effort has been made to showcase the ability of one of the systems to be adaptable for the three vascular procedures with or without minor modification. For example, the CorPath^®^ GRX robotic system has been tested for R-PCI, R-PVI, and R-NVI procedures [73,74,75]. Hence, it can be classified as a multi-specialty vascular robotic system. R-PCI, R-PVI, and R-NVI are classified as endovascular interventions. These procedures use flexible tools such as catheters, guidewires, balloons, and stents, which are navigated to the site of the vessel lesion by VIR systems. On the other hand, robot-assisted electrophysiological procedures have distinct anatomical objectives and a procedural flow that is different from the abovementioned vascular procedures; therefore, electrophysiological-based interventional robots (EPIRs) are mostly single-specialty systems with design configurations that allow their usage for cardiac ablation with catheter-tip deflection and distal-force sensing, catheter steerability, magnetic navigation, and advanced cardiac mapping, an essential consideration to control and navigate the steerable catheter to a desired position and orientation compared with vascular procedures [108,109,110,111]. This is applicable for the treatment of cardiac arrhythmia and atrial flutter. However, the review of literature in this study focuses on endovascular interventions, and profound description of electrophysiological interventions is reported in other literature [112].

**Figure 5:**
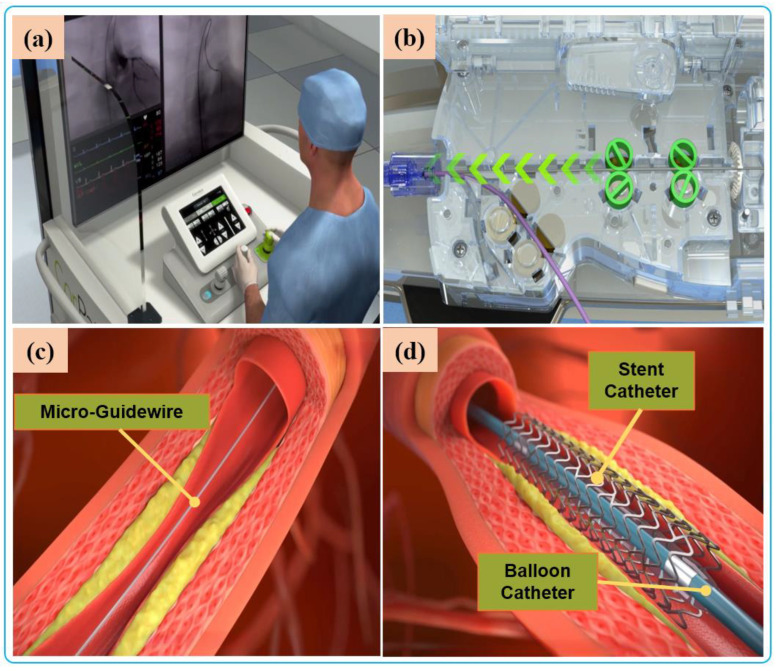
Robot-assisted procedures: (**a**) The interventionalist remote controls the robot on the console. (**b**) The robot delivers guidewires and catheters under the remote operation of the interventionalist. (**c**) Micro-guidewire passes the lesion site. (**d**) The stent is released to immobilize the lesion.

Robot-assisted endovascular interventions are performed in two stages. The first stage is the manual procedure. The main task of the manual procedure is to provide a stable arterial access for the flexible surgical tool, followed by robotic navigation, which is usually performed by a doctor or assistant. The second stage is the robot-assisted procedures wherein the interventionalist remotely controls the robot to deliver guidewires and then the balloons and stent catheters with the aid of 2D real-time image data to the location of the vascular lesions. The two stages are illustrated in Figure 4 and Figure 5, respectively.

**Figure 6:**
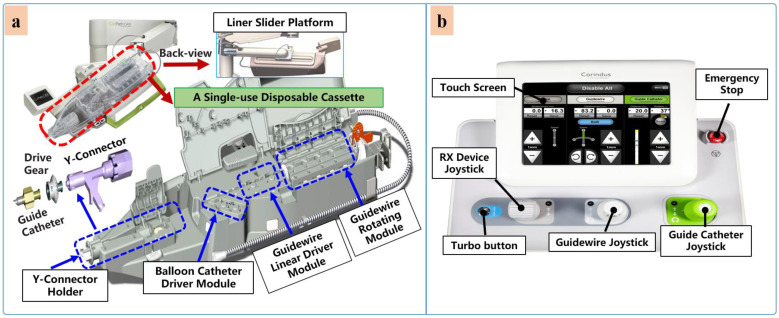
Commercial robotic system CorPath^®^ GRX. (**a**) Bedside-instrument delivery mechanism includes an extended-reach arm and a single-use disposable cassette. (**b**) Robotic joystick controller for the CorPath^®^ GRX system.

Currently, commercial EVIRs specifically include the Corindus CorPath series, Hansen’s Magellan system, and Robocath’ s R-One robot system. Based on the robotic navigation system developed by Beyar et al. [23], Corindus developed the CorPath^®^ 200, which was the first robotic system to receive an FDA approval for coronary interventions, in 2012. Subsequently, the CorPath^®^ GRX system was approved by the FDA in 2016 to serve patients’ vascular needs with physicians’ opinions. The second-generation CorPath^®^ GRX robotic system consists of an extended reach arm, a single-use disposable cassette, and a lead-shielded robotic-control workstation. As shown in Figure 6, this system can simultaneously manipulate the guidewire, guide–catheter balloon, and stent catheter. In addition, the precision and automation of the robot have been enhanced by the addition of “Dotter,” “Constant Speed,” “Active Device Fixation (ADF)” and “Auto Rotate-on-Retract” functions [113,114,115,116]. Overall, the robotic system is compact and could facilitate simplified manipulation of endovascular tools through the control work station; in addition, the drive cassette is a sterile disposable part that can be easily sterilized. However, the disadvantages are that it can only operate one set of guidewire and catheters, which is not fully capable for treating complex lesions, coupled with the high cost of consumables and the lack of distal force feedback [117,118]. The Hansen Magellan^TM^ Robotic system is a remotely steerable catheter system that is based largely on the original Sensei^®^ system but with significant modifications. It is composed of a remote device manipulator, physician workstation, and robotic catheter [76]. The remote device manipulator consists of two manipulators for operating flexible endovascular tools, and a drive friction belt that is designed for stabile tool delivery and distal tip control of the flexible endovascular tools, i.e., catheters and guidewires [16]. The Magellan system is designed for multi-specialty peripheral vascular procedures. To its merit, it is equipped with a self-developed controllable bending catheter. This facilitates smooth access to complex small peripheral vessels, provides rock-solid stability for guidewire delivery, and reduces the risk of vascular injury caused by contact and friction with the vessel wall [119]. However, the system’s disadvantage includes the need to manually place interventional devices (balloons, stents) after the surgical access has been established, the high cost of the robotic system, and the lack of haptic feedback [120].

Beyond the commercial levels, many research teams have conducted research on key technologies of robot-assisted vascular interventions. These are mostly found for force or haptic feedback, operation-safety strategies, and multi-device collaborative-delivery technology. Several VIRs have been developed within the research domain for these key technologies [121].

The ability of the robotic system to simultaneously manipulate multiple guidewires and catheters during the treatment of complex coronary lesions (type B2/C) is a key technology that is urgently needed in the cath lab and the focus of some existing studies. The treatment of complex lesions relies on the cooperative operation of multi-instruments. To achieve this, Ref. [122] designed two bionics to deliver both the catheter and guidewire simultaneously. The delivery of the guidewire is realized by the axial reciprocating motion, and the rotary motion is realized by three rollers that clamp the guidewire to each other and rotate in the same direction and speed. This device can realize the clamping and rotation of two guidewires. Only the crank rocker is interchanged to another set of rollers when switching between the guidewires. Although this mechanism can achieve multi-instrument movement, the volume and weight of the mechanism are usually large, and the clamping-wire part will form multi-segment bending, which may damage the instrument. In another study, Cha et al. [27] used a combination of linear reciprocating and rotating gear teeth to complete catheter translation and rotation, and used the combination of friction wheel and gear to drive the guidewire translation and rotation [82]. The authors carried out an in-vivo study to verify the robotic-system adaptability and functions for multi-instrument handling, control, and navigation [81]. The device has the advantages of easy disassembly and sterilization of component parts.

## 3. Clinical Adoption and Evaluation

Robot-assisted vascular interventional therapy has achieved satisfactory and fruitful results in early clinical trials in human subjects. In 2006, Beyar et al. used the RNS for the first time to conduct a robot-assisted PCI interventional surgery trial for 18 patients and successfully completed the guidewire crossing through the lesion in 17 patients, with a clinical success rate of 100%, a technical success rate of 94%, and an overall surgical success rate of 83% [23]. This is a milestone towards the maturity of vascular interventional surgery robots. With the continuous improvement of vascular intervention robots, surgeons can begin to perform robot-assisted vascular intervention surgery in many parts, such as in interventional cardiology, peripheral vascular surgery, neurovascular surgery, cardiac electrophysiology, and so on.

In the clinical report on the evaluation of R-PCI, Granada et al. reported the first human trial of the CorPath^®^ 200 robotic system in the percutaneous coronary artery [21]. The clinical trial involved performing robot-assisted delivery and manipulation of coronary guidewires, balloons, and stents in eight patients, and they evaluated the safety and feasibility of the system. The results showed that compared with manual surgery, the radiation damage of doctors was reduced by 97%. The technical success rate of the robot system was 97.9%, and there were no equipment-related complications. Furthermore, Weisz et al. evaluated the safety and clinical efficacy of CorPath^®^ 200 R-PCI in the PRECISE (Percutaneous Robotically Enhanced Coronary Intervention) clinical trial in 2013 [22]. A total of 164 subjects underwent in R-PCI clinical trials, of which 112 patients (68.3%) had A or B1 lesions, and the rest were type B2 (18.9%) or type C (12.8%). The maximum lesion length was 24 mm. In the final trial, 160 patients (97.6%) were clinically successful, and the amount of radiation received by the operator in the cockpit was 95.2% lower than that measured on the operating table (0.98 vs. 20.6 µGy), demonstrating that R-PCI addresses some of the occupational hazards for interventionalists without affecting operation performances and safety for patients. Mahmud et al. [29] recruited 315 patients with complex lesions of type B2 and C in the CORA-PCI (Complex Robotically Assisted Percutaneous Coronary Intervention) study and divided them into two control groups: R-PCI and manual percutaneous coronary intervention (M-PCI). The results showed that the clinical success rate of R-PCI was 99.1% equal to that of M-PCI. This study demonstrated the feasibility, safety, and high technical success of R-PCI for the treatment of complex coronary disease. Subsequently, Smitson et al. [73] reported the first human clinical trial of the second-generation robotic-assisted system CorPath^®^ GRX in the treatment of complex coronary artery disease, with a clinical success rate of 97.5%, reflecting the safety and effectiveness of CorPath^®^ GRX in the treatment of complex coronary artery disease.

In the clinical report of robot-assisted PVI, Mahmud et al. reported the feasibility and safety of CorPath^®^ 200 in the treatment of peripheral arterial diseases [32]. The study enrolled 20 subjects with primarily Rutherford class 2 to 3 (90%) symptoms and treated 29 lesions. The technical success rate, safety, and clinical success rate of the system were 100%. There were no adverse accidents related to the robot system, demonstrating the feasibility and safety of using a robotic-assisted platform for performing peripheral arterial revascularization and leading to FDA approval of the device for peripheral interventions. In order to facilitate the robot system for NVI, CorPath^®^ GRX makes some modifications, including the ADF, which fixes the guidewire during microcatheter movement, and the addition of guide-catheter movement. Some clinical studies described a satisfactory procedure performance of this modified robot in NVI. Weinberg et al. [31] verified the feasibility and safety of transradial carotid-artery stenting (TRCAS) assisted by the CorPath^®^ GRX robot. After comparing the robotic and manual procedure, the results showed that there were no significant differences in baseline characteristics, contrast-agent dose, radiation exposure, catheter replacement, technical success, or transfemoral artery-conversion rate, and no technical or approach complications between the two groups, demonstrating that RA-TRCAS is feasible, safe, and effective. Potential concerns are the lack of effective force feedback for current robots compared with manual procedures and the long learning cycle for robot-manipulation skills. Perera et al. [123] published a clinical report stating that arch-catheter placement reduces cerebral embolization during thoracic endovascular aortic repair (TEVAR) with the Magellan robot. A comparison of the robot-assisted and manual procedures showed that cerebral embolization with robotic catheter placement was better.

In clinical trials of robot-assisted electrophysiological ablation, several retrospective studies on the ablation of atrial fibrillation and atrial flutter with the Sensei X-ray robotic navigation system have found that it is feasible to use a remote-controlled robot system for cardiac mapping and radiofrequency ablation of atrial fibrillation and atrial flutter. This can significantly increase the duration of atrial fibrillation ablation with a low recurrence rate of atrial fibrillation [124]. Other studies evaluated the feasibility, efficacy, and safety of cavo-tricuspid isthmus ablation with the Amigo remote catheter system in patients with typical atrial flutter and in mapping the right side of the heart. The results from the studies demonstrated the efficacy and safety of Amigo in mapping the right side of the heart, and that it can safely and effectively perform cavo-tricuspid isthmus ablation for typical atrial flutter [125]. Although the VIR system has been proven to be feasible and effective in the clinic, there are still many limitations of the robot system. Firstly, force perception or tactile feedback cannot be provided to interventionists, but is essential when navigating small-diameter vessels. Secondly, the current robotic system does not support wired coronary intervention. Although the currently available coronary-artery robotic system allows the operation of airbags, two important steps in the operation are still performed manually: obtaining the arterial passage and operating the guiding catheter. In the context of limited resources and costs, it is important to point out that robot-assisted PCI can be associated with prolonged procedural time compared with traditional manual PCI [126]. A summary of the main clinical trials and studies on related vascular interventional robotic systems is presented in Table 2.

## 4. Discussion and Outlook

Robot-assisted endovascular interventions in different vascular tissues of human anatomy are becoming more acceptable because robot-assisted technology has the significant potential to enhance the accuracy and safety of the procedures, minimize health risks, and deliver measurable benefits to the patients and the interventionalist. Despite the above-mentioned progress, the following technical challenges need to be further studied to make VIRs more widely adopted in tertiary-care centers.

- Cooperative Driving Mechanisms of Multiple Instruments

With the iterations and innovations in VIR technology, the clinical limitations are being reduced. For example, the CorPath^®^ GRX system, the second generation of the Corindus robotic platform, increased the guide-catheter manipulation, guidewire retraction, and other functions; significantly enhanced the ability of robot-assisted treatment of complex lesions; and expanded the scope of robot-assisted vascular interventional therapy. Nonetheless, the current commercially available VIRs only operate one coronary guidewire and locate one balloon and stent at the same time, which cannot provide sufficient support for the balloon and stent in complex blood vessels, and cannot manipulate over the wire equipment [115]. Typically, the robotic system is fitting for carrying out simple lesion operations, whereas the success rate for complex procedures that require two guidewires to operate together is not high [114]. In fact, in the interventional treatment of cardio-cerebrovascular diseases and complex lesions accounts for more than 50%, including various chronic complete occlusions, severe calcification, and bifurcation lesions. Thus, there is a need for a multi-instrument robotic delivery system compatible with conventional endovascular interventions tools. This also requires improved software- and hardware-control modules for multi-instrument control to further reduce some burdens surgeons undertake during complex lesion interventions.

- Perception and Feedback

In endovascular interventions, experienced physicians rely on the weak haptic feedback from the handheld instruments to ascertain the guidewire/catheter state within the vessels and to minimize operative risks. However, existing commercial surgical robots have not yet found an optimal solution for mechanical-force feedback and haptic perception. Therefore, surgeons are unable to perceive the guidewire resistance during procedures, which makes remote teleoperation a challenge for surgeons. It is one of the main factors that limit the precise control and widespread use of VIRs [128]. Although researchers have tried different ways to solve this challenge, the technology is not yet mature and stable. To realize force feedback, sensor-based direct-force measurement technology and the model-based force-prediction method are promising areas attracting in-depth research and application. In terms of tactile perception technology, a small number of commercial devices can transmit high-fidelity feedback to surgeons, giving surgeons a clinical sense of touch, but this heterogeneous way of tool manipulation violates doctors’ traditional catheterization habits and makes it more difficult to learn. The development of tactile-perception function in VIR is still in its infancy, and we believe that the development of an isomorphic force–tactile manipulation platform will open up a more intuitive and applicable frontier direction for endovascular intervention.

- Teleoperation Setup and Automation Surgeries

In endovascular procedures, teleoperation is still the most effective standard method for performing surgery [129]. Through remote teleoperation, interventionalists can perform vascular procedures thousands of miles from the patients in space, which helps to alleviate the uneven regional distribution of health-care resources. The continuous maturity, stability, and wider coverage of the communication network provides a technical guarantee for the feasibility and safety of remote surgery. Clinical trials have proven that remote robot-assisted vascular interventions can provide solutions and guidance to emergency centers lacking cardiovascular specialists in remote and underdeveloped areas [127]. However, ensuring visual and tactile high fidelity and low transmission delay is still the main research challenge limiting remote telesurgery.

The development goal of medical robots is to replace doctors to complete some surgical work independently, and it is divided into six levels according to the automation level of medical robots in Ref. [130]. At present, VIRs are in level 1 and advancing towards level 2. Compared with other surgical robots, VIRs have a natural advantage in the development of surgical automation, because the operation inside the vascular lumen has a certain level of regularity and repeatability. However, due to the flexibility of the endovascular tools and the complex dynamic environment in the body, it is still a great challenge to accurately predict and compensate for the motion of the device to realize the advanced automation levels in VIRs. In addition, ethical and legal concerns related to patient safety are also issues that challenge the introduction of increased automation in VIRs. Therefore, the development of higher-level automation in future VIRs needs to overcome the existing limitations.

## 5. Conclusions

The introduction of robotic systems for endovascular procedures has brought present gains for both the patients and the interventionalists. However, there is hope of achieving further progress by finding reliable solutions to existing challenges within this domain, thereby enhancing the measurable benefits to patients opting for robot-assisted interventions. Typically, vascular interventions such as catheterization are now more precise, and the robotic interfaces are also playing important roles in reducing the operational hazards that surgeons experience during the interventions while patients now experience improved and more efficient surgical outcomes. However, other key factors that limit the promotion of these technologies or products and restrict the development of robotic technology in interventional cardiology have been outlined in this study. This study has analyzed and summarized the technical and clinical progress of robot-assisted endovascular interventions, along with an overview of some of the pertinent challenges hindering their wider adoption. In the future, we hope that more studies will spring out to tackle the setbacks highlighted in this review study.

## Figures and Tables

**Table 2:** Summary of main clinical trial studies on related vascular interventional robotic systems.

Paper	Device	Year	Intervention	Treated Lesions	Technical Accuracy (%)	Clinical Accuracy (%)
Mahmud et al. RAPID [32]	CorPath^®^ 200	2016	R-PVI	20	100	100
Mahmud et al. CORA-PCI [29]	CorPath^®^ 200	2017	R-PCI	157	91.7	99.1
Perera et al. [123]	Magellan^TM^	2017	R-TEVAR	11	N/A	100
Smitson et al. [73]	CorPath^®^ GRX	2018	R-PCI	40	90	97.5
Patel et al. REMOTE-PCI [127]	CorPath^®^ GRX	2019	Tele-PCI	5	100	100
Weinberg et al. [31]	CorPath^®^ GRX	2020	RA-NVI	13	100	100
